# Supplementary material for: Chromosome Painting in Cultivated Bananas and Their Wild Relatives (Musa spp.) Reveals Differences in Chromosome Structure
Source: Int J Mol Sci. 2020 Oct 24;21(21):7915. doi: 10.3390/ijms21217915 (PMC7672600; doi:10.3390/ijms21217915)
Supplement: Supplementary file 1 [file ijms-21-07915-s001.zip › Suppl. Tab. S1.pdf]

Supplementary Table S1.

	Diploid accessions														Triploid accessions								
	**M. acuminata ssp. (AA)**								**Mchare cultivars (AA)**			**M. balbisiana (BB)**		**M. schizocarpa (SS)**	**AAA**				**AAB**			**ABB**	
															**Cavendish**	**Gros Michel**	**EAHB**		**Plantains**			**Pelipita**	**Saba**
**Chromosome change**	*malaccensis* **'Pahang'****	*banksii* **'Bansksii'**	*microcarpa* **'Borneo'**	*Zebrina* **'Maia Oa'**	*burmannicoides* **'Calcutta 4'**	*burmannica* **'Tavoy'**	*siamea* **'Pa Rayong'**	***P. lilin***	**Huti White**	**Huti (Shumba nyeelu)**	**Ndyali**	**Tani****	**Pisang Klutuk Wulung**	**Schizocarpa****	**Poyo**	**Gros Michel**	**Imbogo***	**Kagera**	**3 Hands Planty**	**Amou***	**Obino l'Ewai**	**Pelipita**	**Saba sa Hapon**
**3S/8L + 8S/3L**				••		•			•	•	•				•	•	••	••					
**9S/1L + 1L/9S**					••	•	••																
**8L/2L**					••	•	••																
**7S/8S + 7L/8L**						•																	
**3L/4S**							•																
**4L/1L + 1L/4L**								•	•	•	•				•	•							
**7S/1L + telocentric 7L**															•	•							
**1L/3L (B genome specific)**												••	••						•	•	•	••	••
**7L/1L + missing 7S and 1S**																	•						
**missing chromosome 2**																				•			

*** aneuploid clones**

**** data from Šimoníková *et al*. (2019)**
