# Supplementary material for: Chromosome Painting in Cultivated Bananas and Their Wild Relatives (Musa spp.) Reveals Differences in Chromosome Structure
Source: Int J Mol Sci. 2020 Oct 24;21(21):7915. doi: 10.3390/ijms21217915 (PMC7672600; doi:10.3390/ijms21217915)
Supplement: Supplementary file 1 [file ijms-21-07915-s001.zip › Supplementary Materials.docx]

Supplementary Materials


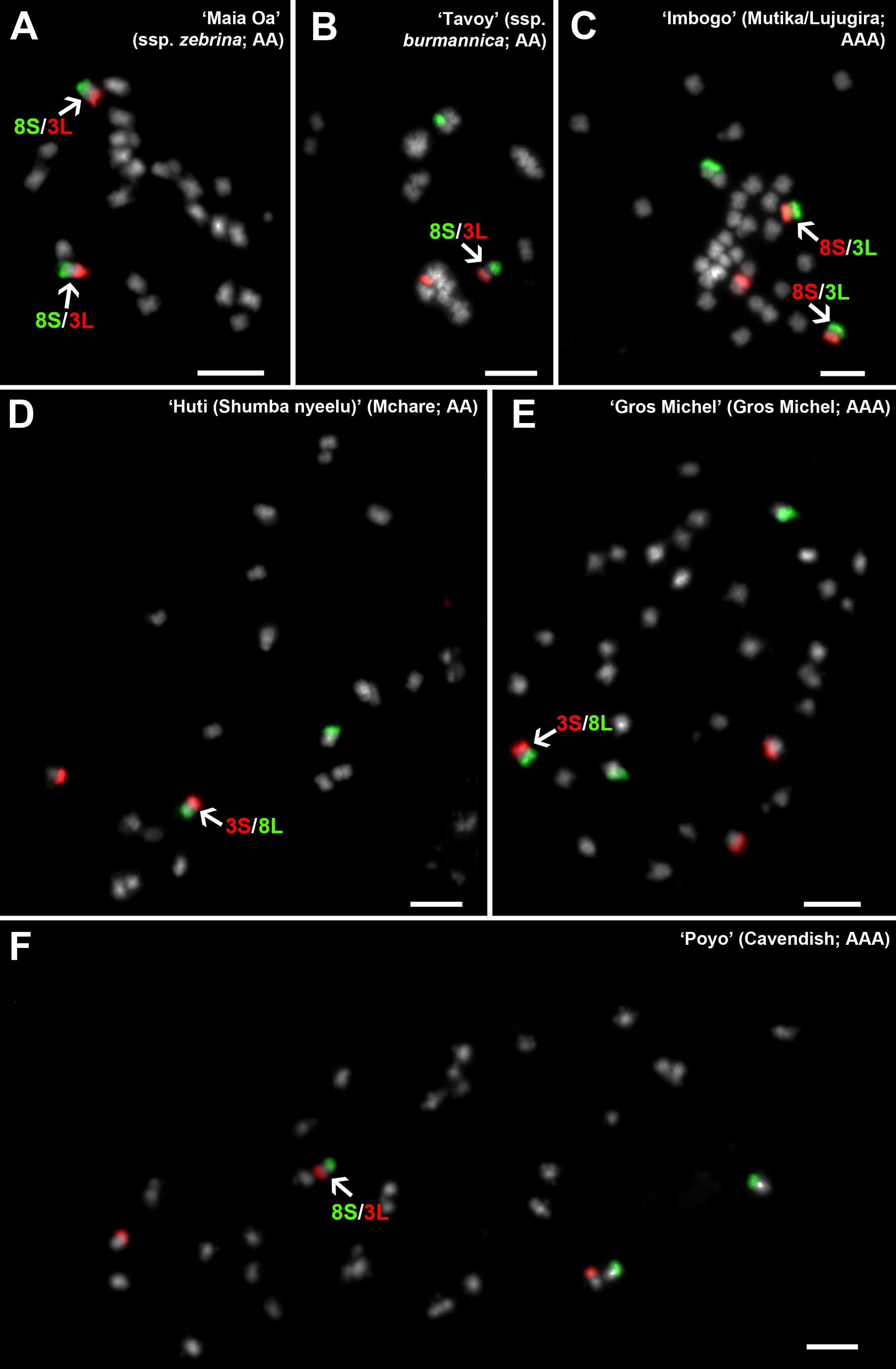


**Supplementary Figure S1.** Examples of oligo painting FISH on mitotic metaphase plates of six *Musa* accessions: (A) ‘Maia Oa’ (ssp. *zebrina*, 2n=2x=22, AA genome), probes for long arm of chromosome 3 and short arm of chromosome 8 were labeled in red and green, respectively; (B) ‘Tavoy’ (ssp. *burmannica*, 2n=2x=22, AA genome), probes for long arm of chromosome 3 and short arm of chromosome 8 were labeled in red and green, respectively; (C) ‘Imbogo’ (Mutika/Lujugira clone, 2n=3x - 1= 32, AAA genome): probes for long arm of chromosome 3 and short arm of chromosome 8 were labeled in greed and red, respectively; (D) ‘Huti (Shumba nyeelu)’ (Mchare clone, 2n=2x=22, AA genome), probes for short arm of chromosome 3 and long arm of chromosome 8 were labeled in red and green, respectively; (E) ‘Gros Michel’ (Gros Michel clone, 2n=3x=33, AAA genome), probes for short arm of chromosome 3 and long arm of chromosome 8 were labeled in red and green, respectively; (F) ‘Poyo’ (Cavendish clone, 2n=2x=33, AAA genome), probes for long arm of chromosome 3 and short arm of chromosome 8 were labeled in red and green, respectively. Chromosomes were counterstained with DAPI (light grey pseudocolor). Arrows point translocation chromosomes. Bars = 5 µm.


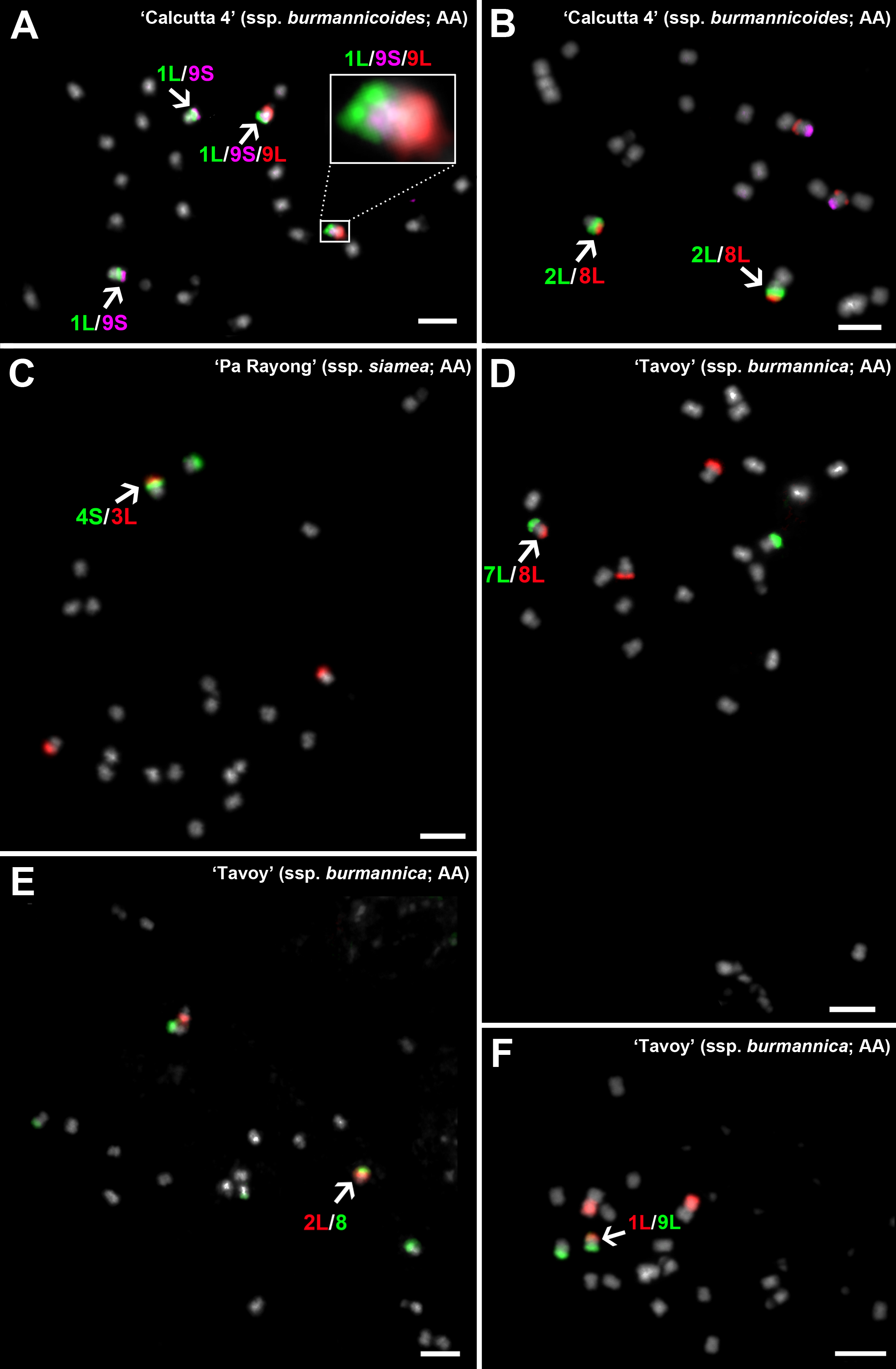


**Supplementary Figure S2.** Examples of oligo painting FISH on mitotic metaphase plates of six *Musa* accessions: (A) ‘Calcutta 4’ (ssp. *burmannicoides*, 2n=2x=22, AA genome), probes for long arm of chromosome 1, long arm of chromosome 9 and short arm of chromosome 9 were labeled in green, red and purple, respectively; (B) ‘Calcutta 4’ (ssp. *burmannicoides*, 2n=2x=22, AA genome), probes for long arm of chromosome 2, long arm of chromosome 8 and short arm of chromosome 8 were labeled in green, red and purple, respectively; (C) ‘Pa Rayong’ (ssp. *siamea*, 2n=2x=22, AA genome), probes for long arm of chromosome 3 and short arm of chromosome 4 were labeled in red and green, respectively; (D) ‘Tavoy’ (ssp. *burmannica*, 2n=2x=22, AA genome), probes for long arm of chromosome 7 and long arm of chromosome 8 were labeled in green and red, respectively; (E) ‘Tavoy’ (ssp. *burmannica*, 2n=2x=22, AA genome), probes for long arm of chromosome 2 and chromosome 8 were labeled in red and green, respectively; (F) ‘Tavoy’ (ssp. *burmannica*, 2n=2x=22, AA genome), probes for long arm of chromosome 1 and long arm of chromosome 9 were labeled in red and green, respectively. Chromosomes were counterstained with DAPI (light grey pseudocolor). Arrows point translocation chromosomes. Bars = 5 µm.


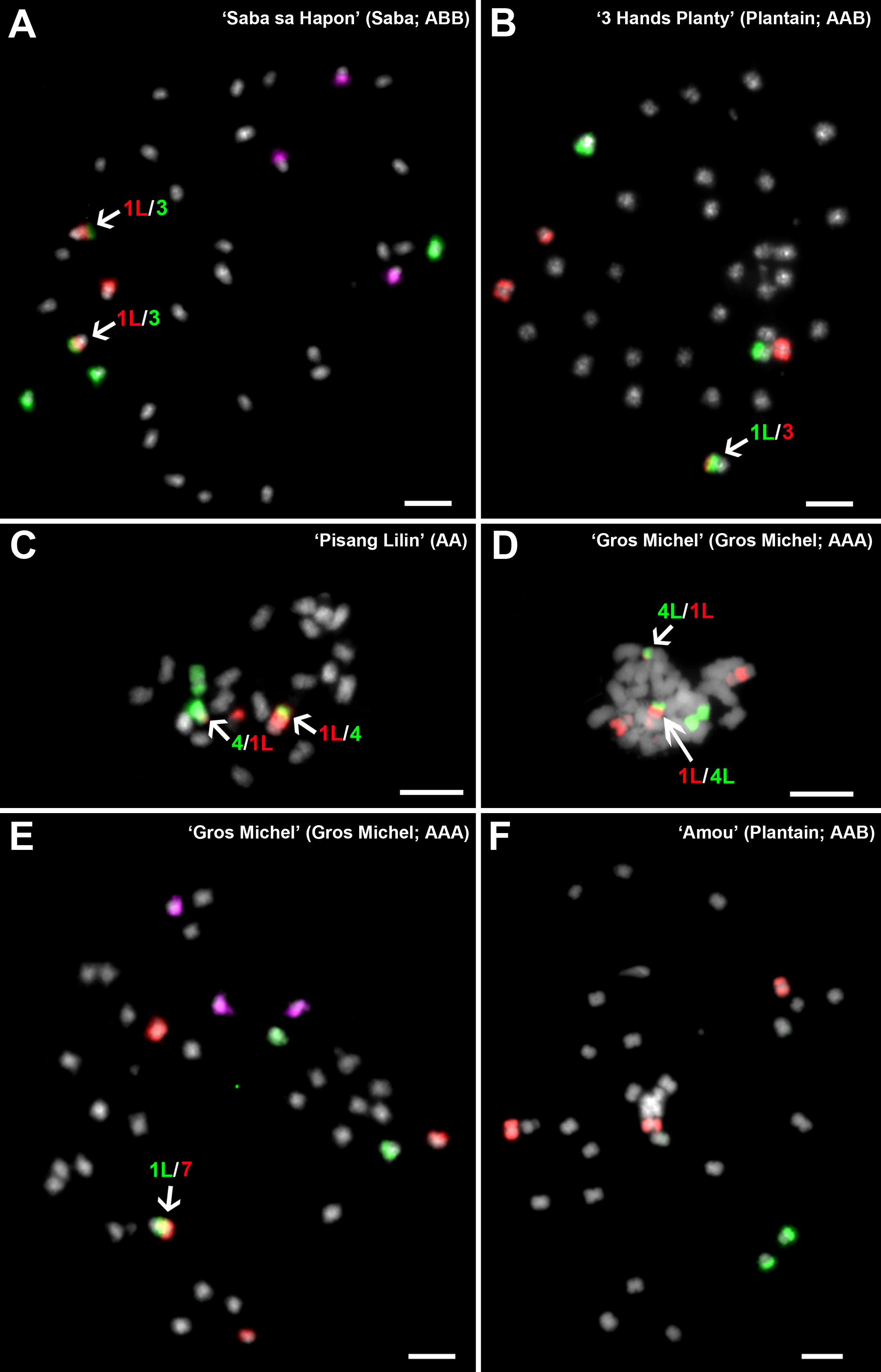


**Supplementary Figure S3.** Examples of oligo painting FISH on mitotic metaphase plates of six structurally heterozygous *Musa* accessions: (A) ‘Saba sa Hapon’ (Saba clone, 2n=3x=33, ABB genome), probes for long arm of chromosome 1, long arm of chromosome 2 and chromosome 3 were labeled in red, purple and green, respectively; (B) ‘3 Hands Planty’ (Plantain clone, 2n=3x=33, AAB genome), probes for long arm of chromosome 1 and chromosome 3 were labeled in green and red, respectively; (C) ‘Pisang Lilin’ clone (2n = 2x = 22, AA genome), probes for long arm of chromosome 1 and chromosome 4 were labeled in red and green, respectively; (D) ‘Gros Michel’ (Gros Michel clone, 2n=3x=33, AAA genome), probes for long arm of chromosome 1 and long arm of chromosome 4 were labeled in red and green, respectively; (E) ‘Gros Michel’ (Gros Michel clone, 2n = 3x = 33, AAA genome), probes for long arm of chromosome 1, long arm of chromosome 2 and chromosome 7 were labeled in green, purple and red, respectively; (F) ‘Amou’ (Plantain clone, 2n = 3x – 1 = 32, AAB): probes for long arm of chromosome 2 and chromosome 4 were labeled in green and red, respectively. Chromosomes were counterstained with DAPI (light grey pseudocolor). Arrows point translocation chromosomes. Bars = 5 µm.

**
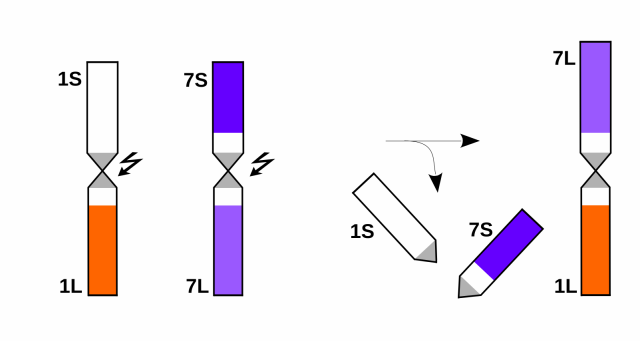
 Supplementary Figure S4: Robertsonian translocation as a mechanism leading to structural chromosome change (formation of a translocation chromosome 7L.1**L) and aneuploidy (loss of the short arms of chromosomes 1 and 7) in East African Highland Banana clone ‘Imbogo’.


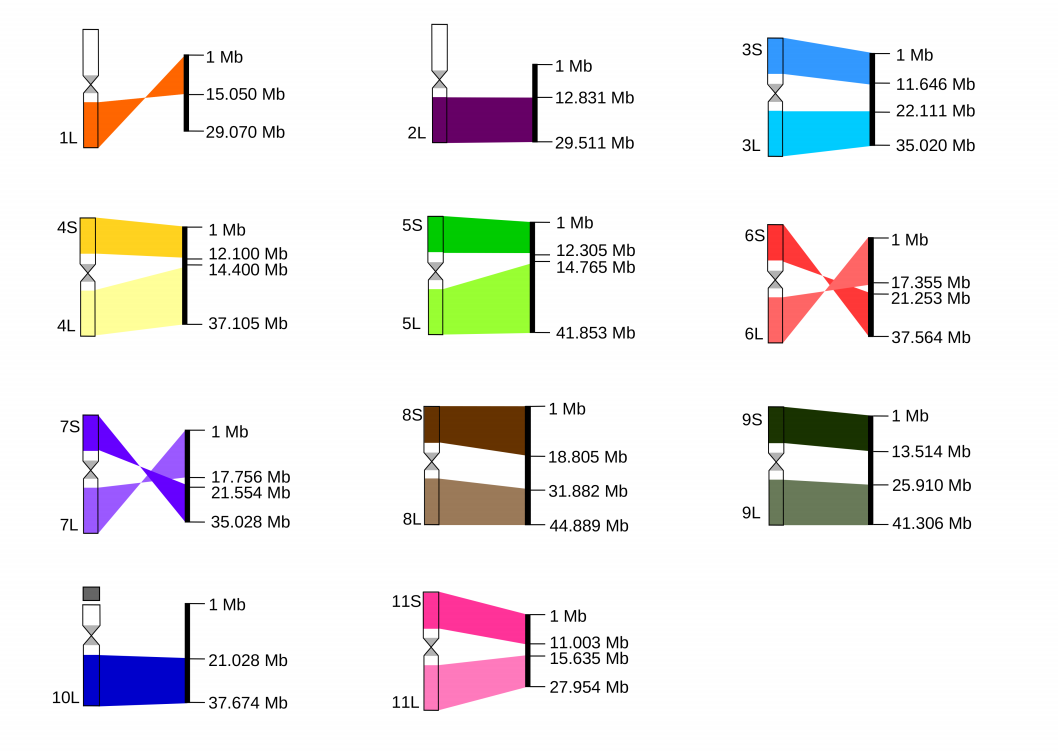


**Supplementary Figure S5: Anchoring of chromosomes to *M.*** *acuminata* ‘DH Pahang’ pseudomolecules which were used to develop oligo painting probes. Note that in the reference genome assembly, pseudomolecules 1, 6, 7 are oriented inversely to the traditional way karyotypes are presented, where the short arms are on the top.
